# Do Stand-Biased Desks in the Classroom Change School-Time Activity and Sedentary Behavior?

**DOI:** 10.3390/ijerph16060933

**Published:** 2019-03-15

**Authors:** Ann M. Swartz, Nathan R. Tokarek, Krista Lisdahl, Hotaka Maeda, Scott J. Strath, Chi C. Cho

**Affiliations:** 1 Department of Kinesiology, University of Wisconsin-Milwaukee, Milwaukee, WI 53211, USA; ntokarek@uwm.edu (N.R.T.); sstrath@uwm.edu (S.J.S.); 2 Center for Aging and Translational Research, University of Wisconsin-Milwaukee, Milwaukee, WI 53211, USA; hotaka.maeda@gmail.com (H.M.); chocc@uwm.edu (C.C.C.); 3 Department of Psychology, University of Wisconsin-Milwaukee, Milwaukee, WI 53211, USA; medinak@uwm.edu

**Keywords:** actigraphy, children, control group, sedentary lifestyle, standing, intervention, school

## Abstract

The purpose of this study was to determine the effect of stand-biased desks on the physical activity and sedentary behavior of third, fourth and sixth grade students across the school year. *Methods*: This within classroom crossover design study used teacher-determined allocation for seating within each classroom. Half of the students used a stand-biased desk and half used a sitting desk. Five-day hip-worn accelerometer assessments were completed at baseline and at the end of each nine-week intervention period. A mixed effects model was used to determine the differences in the percentage of time spent active and sedentary. *Results*: A total of 22, 36 and 41 students in 3rd, 4th and 6th grades, respectively, completed this study (57.1% male, 79.3% White). Regardless of the desk type, students became more sedentary (*p* < 0.001) and less active (*p* < 0.001) in the classroom as the school year progressed. After controlling for baseline activity, there was a significant interaction between the type of desk and time (*p* = 0.029). Students who spent a higher percentage of their classroom time sedentary engaged in less sedentary behavior when using a stand-biased desk compared to the traditional desk. *Conclusion*: The standing desk intervention was effective in mitigating the increase in sedentary behavior for those who started the school year more sedentary.

## 1. Introduction

Movement is very important for the growth and development of children. Children need to move often to develop strength, balance, coordination and their sensory system. Furthermore, movement helps to reduce anxiety and stress and increase self-esteem. However, a major proportion of a child’s environment is designed to encourage sitting. Children sit during meals, on their way to school, during school, doing homework after school, playing video games, watching television and using their phone/tablet. These multiple opportunities to be sedentary are seen in data obtained from objective movement sensors (accelerometers), which show that children aged 6–11 years sit for approximately 40–45% of their waking day or approximately 6 h/day [1], while self-reported data suggest that the time spent sedentary is almost double the objective estimates [2,3,4].

This excessive amount of sitting has a negative impact on the health and wellbeing of children. Sedentary behavior in children is adversely linked to unfavorable body composition and biomarkers for cardiovascular and metabolic disease risk, including high blood pressure, high cholesterol and high insulin resistance. This has also been linked with low fitness levels, reduced self-esteem and poor cognitive development and reduced academic achievement [5,6,7,8,9]. Furthermore, sedentary behavior in childhood has consequences in adulthood as sedentary children tend to become sedentary adults [10,11]. Sedentary behavior in childhood also predicts chronic fatigue syndrome in adults and is associated with a higher risk of being overweight and having dyslipidemia [12,13]. Therefore, altering the child’s environment to promote less sitting and improve their health early in life may increase the likelihood of positive health now and in the future.

Schools provide a unique environment to decrease sedentary behavior for children. Most US children attend school for approximately 6.5 h per day for 180 days each year. However, schools (administrators and teachers) have increased pressure to include more intellectual content and achieve higher standards to ensure adequate funding. Therefore, they are challenged by the desire to incorporate healthy behaviors while meeting the academic requirements for the students [14]. As a result, the time for recess, physical education and other opportunities for movement has diminished to meet the academic requirements necessitated by the state or other governing bodies. This has resulted in high amounts of sitting in the classroom, with accelerometer data having showed that students aged 11–12 years spend 69% of their classroom time sitting [15].

Integrating movement into the classroom is a strategy to promote healthy behaviors but there must still be a time allowance for learning. A number of studies have shown the benefits of standing desks both in a short term period and over the course of a semester, resulting in 17–27% higher energy expenditure during a portion of the school day or the entire school day in first and second grade students using a stand-biased desk versus those using a traditional seated desk [16,17,18]. Furthermore, children increase the proportion of time in school spent standing by 26–31% [19,20] and 21–40 min [21,22] after the introduction of a stand-biased desk. However, the impact of school-based standing desks on accelerometer-determined standing, sitting and physical activity has not been evaluated. Most studies to date have included smaller sample sizes and a limited number of grades or classrooms. Finally, little scientific attention has been given to examining the sustainability of physical activity and sedentary behavior responses to the replacement of sitting desks with standing desks in elementary school children. One recent study evaluated the impact of standing desks in multiple classrooms in multiple schools [23]. In this cluster-randomized trial, three standing desks were placed in each classroom for 6 months. Positive intervention effects were seen in the 18 primary school students: a reduction in sitting time (−26 min/school day), time spent in sitting bouts ≥30 min (−19 min/school day) and an increase in standing time (+26 min/school day) and time spent in standing bouts ≥1 min (+29 min/school day). The lack of additional intervention effects may have been due to the low number of desks in each classroom, thereby resulting in limited exposure to the desks [23]. Furthermore, the small sample size completing the objective assessment of posture necessitates the need for further study. These represent critical knowledge gaps in our understanding of the unique impact of a stand-biased desk intervention for children in the school setting.

The purpose of this study is to determine the effect of stand-biased desks in elementary school classrooms on physical activity and sedentary behavior in elementary school children. Specifically, the aim of this study was to examine the impact of stand-biased versus standard sitting desks on sedentary behavior and activity during the school day in male and female 3rd, 4th and 6th grade students. It was hypothesized that a classroom environmental intervention will reduce sedentary behavior while increasing physical activity levels (movement) during the school day.

## 2. Methods

### 2.1. Overview and Participants

This study employed a within classroom crossover design, with teacher-determined allocation for seating within each classroom. There were sufficient desks in each classroom for 50% of the students to use a stand-biased desk and 50% to use a sitting desk, so all students, regardless of involvement in the study or not, were assigned to a desk (sitting or standing) by their teacher prior to enrollment in the study. Desks were positioned so that all students would be seated during baseline assessments. After the baseline assessments were complete, the students in the Stand-Sit group used a stand-biased desk (standing desk with a stool, for the option of sitting) in their classroom for nine weeks during the fall (September) to winter (December) period before using a sitting desk for nine weeks in the winter (January) to spring (April) period. The Sit-Stand group used a sitting desk in their classroom for nine weeks (fall/September to winter/December) before using a stand-biased desk for nine weeks (winter/January to spring/April). The within classroom design was selected to minimize the influence of different teachers on classroom behavior [24].

Teachers and the elementary school principal were approached about the study during the spring prior to the intervention. Eleven teachers expressed interest: one second grade teacher, one third grade teacher, two in multiage (third and fourth grade combined) classrooms, two fourth grade teachers and three sixth grade teachers. Classrooms in the multiage, fourth grade and sixth grade were selected because the entire grade was accounted for by these classrooms and therefore, when any switching between classrooms occurred, children would be able to use the same type of desk (sitting or standing) that was assigned to them in their classroom. Participating teachers received three $50 gift cards for participation, one after each assessment period (baseline, post I, post II), to a total of $150.

Participants in selected classrooms were recruited during the fall registration day for the school district through informational handouts. Interested participants (parents, teachers and students) were invited to a presentation of the proposed study during the school and classroom orientation, where all study procedures were explained and any questions answered. Willing participants were consented (parents and teachers) and assented (children) by the second week of the school year. All procedures were approved by the university institutional review board (UWM IRB #17.019).

This study was designed to evaluate the effect of an environmental intervention on sedentary behavior and activity throughout the day. In each participating classroom, half of the traditional sitting desks were replaced with stand-biased desks and no further adjustments to the classroom or curriculum were implemented. Teachers were asked to encourage the students assigned to the stand-biased desks to stand in the classroom. Standing desks were accompanied by a height-matched stool, making the desk/stool combination a “stand-biased” desk. This provided students with the option to stand, but also to sit when needed.

The protocol included assessments at baseline and at the completion of each nine-week period (post I and post II), for a total of three assessment periods. Assessments occurred in fall (September; Baseline; before introduction of the stand-biased desks; weeks 2–4 of the academic year), winter (December; post I) and spring (April; post II). All assessments were completed by trained personnel.

Baseline assessments included (1) anthropometric measurements of height, weight, distance from floor to elbow while standing (to determine appropriate height to set the standing desk) and (2) a 5-day physical activity and sedentary behavior assessment using a hip-worn accelerometer for all waking hours. Post assessment I and II included a 5-day physical activity and sedentary behavior assessment using a hip-worn accelerometer for all waking hours.

### 2.2. Objective Measures of Active and Sedentary Behaviors

Trained researchers handed out a hip worn accelerometry-based motion sensor (Actigraph GT3X+ or wGT3X-BT), to each participating student at the start of the day on Monday of each monitoring week. Researchers ensured the proper fit of all devices (right hip, midline of thigh, upright, secured to a belt) and explained how to wear and use the devices. Additionally, a wear log and directions for use were handed to each child. The wear log asked students to record the time that the monitor was put on in the morning and taken off at night. Additionally, they were asked to record if they took the device off at any other time in the day (time off and on). Children were encouraged to put their wear log in their homework planner to increase compliance. Students wore the devices during all waking hours from the start of school on Monday to the end of the school day on Friday, when monitors were collected by the research staff. Therefore, the accelerometers recorded five full school days and a maximum of three full waking days (Tuesday, Wednesday, Thursday) and two partial days (Monday 8:15–bedtime; Friday waking time–15:25, end of school day) at baseline and Post I. At Post II, vacation days resulted in the accelerometers recording four school days and a maximum of two full waking days. During Week 1 (3rd and 4th graders), this included Monday from 8:15–bedtime; all day Tuesday and Wednesday; and Thursday waking time–15:25 (end of school day). During Week 2 (6th graders), this recorded four full school days and one partial waking day (Tuesday 8:15–bedtime), including all day Wednesday and Thursday and Friday waking time–15:25 (end of school day). Only the data during the school day were used for this analysis.

The Actigraph GT3X+ and wGT3X-BT (Pensacola, FL, USA) are both triaxial accelerometer-based physical activity monitors that provide congruent data. Furthermore, Actigraph accelerometers have been shown to be valid and reliable activity monitors for measuring physical activity and sedentary behavior in children [25,26].

Accelerometers recorded the data at 100 Hz/s to capture the variable movements of children [25]. Data were downloaded onto a laboratory computer and processed using Actilife v6.13.3 without the low-frequency extension (Actigraph, LLC, Fort Walton Beach, FL, USA). Wear time was validated using the Choi algorithm and cross-referenced with the self-reported participant wear logs for accuracy. Sixty or more minutes of accelerometer activity counts at the level of zero was considered non-wear time and was therefore excluded from analysis. To determine the time spent in sedentary behavior and physical activity behavior, vertical plane counts were summed into 15-s epochs and the cutpoints of Evenson et al. (2008) were applied to determine time spent performing sedentary behavior (IA; 0–25 counts/15 s), light- (LPA; 26–573 counts/15 s) and moderate- to vigorous-intensity physical activity (MVPA ≥ 574 counts/15 s) [25].

### 2.3. Accelerometer Data Analysis

Data from the accelerometer were summarized for each of the three assessment periods as minutes of classroom time and percent of classroom time. Specifically, the primary outcomes of interest for this study are the proportion of and total time spent in sedentary behavior (SB), light intensity physical activity (LPA) and moderate- to vigorous-intensity physical activity (MVPA). Data were collected during weekdays when school was in session. Data were averaged over the 5-day assessment periods for baseline, post assessment 1 and 4-day period for post assessment 2. For the analyses that examined classroom time, this only included the time when students were exposed to the standing desks. A valid day for a participant was determined if the wear time during the school day and classroom time was at least 63% of the fixed scheduled time. This criteria was based on the criteria used to determine valid wear time when examining full-day accelerometry to determine average physical activity levels (10 of 16 h [27]). Participants were included in the data if they provided at least 1 day of valid data. For analyses that focus on PA recommendations, a minimum wear time of 10 h/day and 4 days/week was used to calculate whether students met or did not meet national PA recommendations.

### 2.4. Statistical Methods

Normally distributed continuous variables are summarized as mean ± standard deviation (SD) and skewed continuous variables are summarized as median and interquartile range (IQR), respectively. Categorical variables are described as frequency (n) and percentage (%). To determine whether any continuous variable deviated from normality, the Kolmogorov–Smirnov test was used. Specifically, the measures capturing MVPA were found to deviate significantly from normality and thus, a natural-log transformation was used to normalize the data. Between group (Standing vs. traditional desk) comparisons at each measurement point (and within-subject between any 2 measurements points) were performed using *t*-tests (paired *t*-test) or Wilcoxon rank-sum tests (Wilcoxon Signed Rank test) for normal and non-normal data, respectively. For categorical variables, the Chi-square test of association or Fisher’s exact was used. Since it is hypothesized that the intervention will have a positive effect on sedentary behavior and activity, a directional 1-side test with α = 0.05 was applied.

To evaluate the effectiveness of the intervention over multiple observation periods, a mixed effects model was used to determine the differences in the percentage of time spent sedentary and in physical activity when students are using the traditional desk versus standing desk. Specifically, the main factor of interest in the model was the type of desk and the covariates included gender, grade, time and baseline measures of sedentary behavior or PA. Since randomization was not feasible for this study and the students in the two groups were found to be significantly different at baseline with regards to their physical activity and sedentary behavior, the baseline measurements were used as a covariate in the mixed effects model. Specifically, in the model for sedentary behavior, the baseline sedentary behavior was used as a covariate in the model; and in the model for MVPA, the baseline MVPA measurement was used as a covariate in the model. We also included the student’s gender, grade and the time at which the students were assigned to the standing desk as covariates in all models due to these variables having been shown to have an effect on sedentary behavior and physical activity. It is important to note that 2-sided test with α = 0.05 was used to test for significant model effects. All analyses were performed using SAS 9.4 (Cary, NC, USA). The dataset supporting the conclusions of this article is included within the additional files of this manuscript.

## 3. Results

### 3.1. Sample Descriptive

Demographics and Baseline Activity. Two multiage (3rd and 4th combined), two 4th grade and three 6th grade classrooms, each with their own teacher, were included in this study. Ninety-nine students participated in this study, of which 22, 36 and 41 students were in 3rd, 4th and 6th grades, respectively. Approximately 57.1% of the students were male, 79.3% are White and 62.8% reported a household income of $100,000 per year or more. On average, participants had a healthy weight with third graders averaging in the 55th BMI percentile, fourth graders averaging in the 43rd BMI percentile and sixth graders averaging in the 61st percentile, respectively. Overall, based on the accelerometer output, this was an active sample with 70.0% of third graders, 41.2% of fourth graders and 55.6% of sixth graders meeting the physical activity recommendations of 60 min of MVPA per day at baseline, with no significant difference across grades (*p* = 0.115). The results showed that boys tended to be significantly more active than girls in this sample, with 63% of boys and 41% of girls meeting the physical activity guidelines at baseline (*p* = 0.041).

In a typical week, students spent 34.7 h at school, of which the classroom time accounted for a majority of the school time. However, depending on the grade, the amount of time spent in the classroom where they were exposed to the intervention differed. Specifically, the 3rd/4th mixed grade class spent approximately 18.7 h (53.7% of the school week) in the classroom compared to 20.1 h (57.8% of the school week) and 22.5 h (64.8% of the school week) for 4th and 6th grade classroom, respectively. At baseline, when all participating students were seated in a traditional seated desk, approximately 50.5% of the students achieved an average of 30 min or more of MVPA while in their classroom. However, although not significant, there are notable differences in the prevalence of achieving an average of 30 min of MVPA per day during classroom time during the school week across grades. Specifically, 70.0%, 37.1% and 52.5% of the third, fourth and sixth graders (*p* = 0.061) achieved 30 min of MVPA while in the classroom, respectively. Additional information and descriptors of the study sample can be found in Table 1.

### 3.2. Group Comparison at Baseline

For a number of practical classroom related concerns, the random allocation of students to desks was not feasible. Instead, this study utilized a teacher-determined allocation of students in their respective classrooms. There were sufficient desks in each classroom for 50% of the students to use a stand-biased desk and 50% to use a sitting desk so all students, regardless of involvement in study or not, were assigned to a desk (sitting or stand-biased) by their teacher prior to enrollment in the study. This desk assignment process resulted in an imbalance in the number of participants between the two intervention groups at baseline. Furthermore, significant baseline differences between the groups were observed, which is potentially due to the groups not being randomly assigned.

Specifically, the third and fourth grade students assigned to a stand-biased desk for the fall to winter assessment period tended to be more sedentary in the classroom, as assessed by the accelerometer, than students allocated to the traditional sitting desk (3rd: 109.0 vs. 78.5 min sedentary; *p* = 0.005 and 4th: 134.7 vs. 109.6 min sedentary; *p* = 0.021). Accordingly, these third and fourth grade students who were initially allocated to a stand-biased desk also accrued significantly less MVPA during classroom time than students in traditional desk during the fall to winter period (3rd: 14.0 vs. 60.3 min MVPA; *p* = 0.001 and 4th: 5.1 vs. 23.2 min MVPA; *p* = 0.002). Finally, third and fourth graders who are assigned to the stand-biased desk were significantly less likely to accrue an average of 30 min or more of MVPA per day during the school week (3rd: *p* = 0.011; 4th: *p* = 0.033) and have fewer school days where they accumulate 30 min or more of MVPA (3rd: *p* = 0.002; 4th: *p* = 0.032), compared with those in a sitting desk during the fall to winter period. It is important to note that although sixth graders tended to be less active than their third and fourth grade colleagues, no significant differences with regards to sedentary behavior and physical activity during the school day or in classroom time was found between the students assigned to the stand-biased and traditional desks at baseline (Table 2).

### 3.3. Change across Time

Regardless of the desk type, the students became more sedentary (*p* < 0.001) and less active (*p* < 0.001) in the classroom as the school year progressed. Specifically, at the beginning of the school year (September/baseline), students as a whole spent about 61.2% of classroom time sedentary. This increased to approximately 65.4% and 68.8% of classroom time spent sedentary by Post I and II, respectively. Meanwhile, MVPA in the classroom decreased from approximately 4.7% in September/baseline to 3.4% and 2.3% in Post I and II, respectively, and LPA in the classroom decreased from approximately 32.8% in September/baseline to 31.6% and 29.7% in Post I and II, respectively. The 4.2% increase in sedentary behavior and 1.3% decrease in MVPA from baseline to Post I translated into an increase of approximately 8.1 min in sedentary time and a 2.8 min decrease in MVPA. It is important to note that although the changes from baseline to Post I were significant (*p* < 0.05) for both sedentary behavior and MVPA, the changes from Post I to Post II were not significant.

Although the students tended to be more sedentary and engage in less MVPA as the school year progressed, significant differences were observed in the degree of the increase in sedentary behavior and decrease in MVPA based on the type of desk. Specifically, students assigned to the stand-biased desk, on average, demonstrated a smaller increase in sedentary behavior by 2.4% compared to an increase of 6.5% for students in traditional desk from baseline to Post I (*p* = 0.038). Furthermore, whereas student assigned to the standing desk decreased their MVPA by 0.7% from baseline to Post I, students in the traditional desk decreased their MVPA by an average of 5.0% (*p* = 0.001).

### 3.4. Effectiveness of Intervention: Group-by-Time

**Figure 1:**
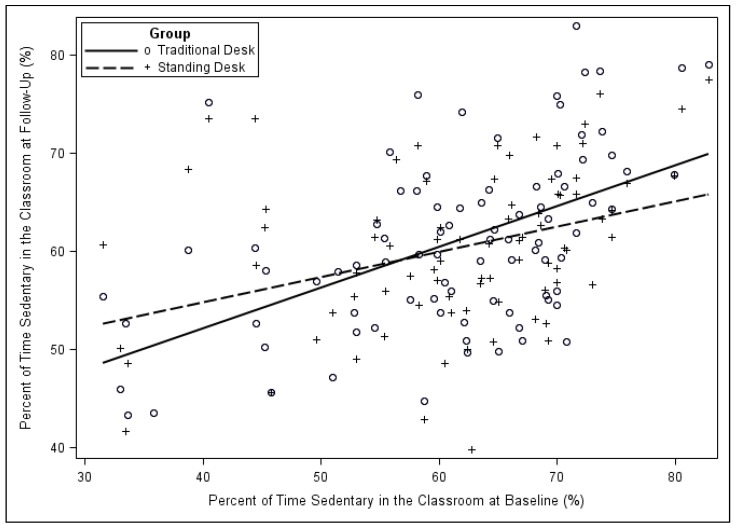
Relationship between sedentary behavior at baseline and follow-up for students using traditional and stand-biased desks. (+ = Standing Desk; ○ = Traditional Desk).

Sedentary behavior Outcome. There was a significant interaction between the type of desk and time (*p* = 0.029) after controlling for baseline activity (see Table 3 and Figure 1). In other words, we found that although the students tended to become more sedentary and their baseline measurement had a significant effect on subsequent sedentary behavior (*p* < 0.001), the standing desk intervention was effective in mitigating the degree of the increase in sedentary behavior. Specifically, for students who spend a higher percentage of their classroom time sedentary, the standing desk was able to reduce the amount of sedentary behavior compared to the traditional desk. However, students who started with a lower level of sedentary behavior (<40% of classroom time sedentary) did not experience the desired impact resulting from the stand-biased desk.

**Figure 2:**
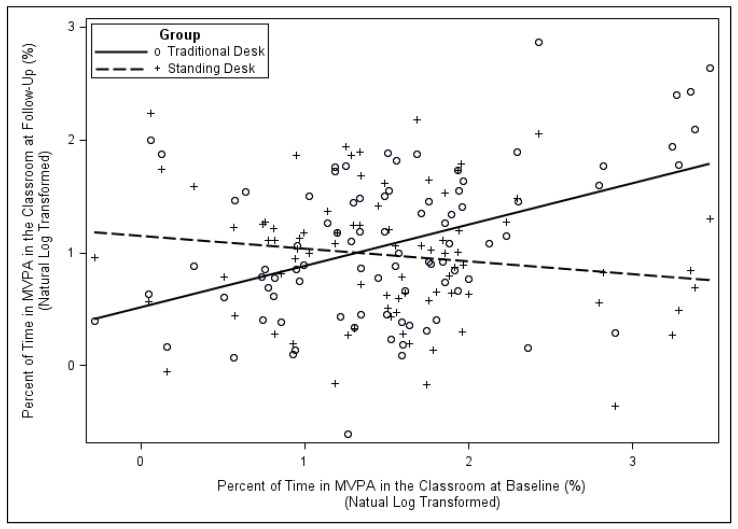
Relationship between MVPA at baseline and follow-up for students using traditional and stand-biased desks. (+ = Standing Desk; ○ = Traditional Desk). Note: As MVPA is not normally distributed, the figure is shown in their natural log transformed scale.

MVPA Outcome. For MVPA in the classroom, the standing desk did not stimulate more activity for those students who were already highly active at baseline. Instead, the benefit of the standing desk on MVPA was seen in students who were not very active at baseline. Specifically, for students who had relatively low MVPA at baseline, their activity when using the stand-biased desk was higher than their activity when using the traditional desk (*p* < 0.0001). This significant type of desk and baseline measure interaction is illustrated by the interacting lines in Figure 2.

LPA Outcome. Finally, the data show that the stand-biased desk intervention did not have a significant effect on LPA (Table 3).

## 4. Discussion

This study was designed to evaluate the effect of an environmental intervention on classroom sedentary behavior and activity. Students were provided with a standing desk and stool (stand-biased desk) or a traditional sitting desk and chair. Teachers were asked to encourage students to stand but no curricular intervention was included. The results of this study show that the elementary school students become more sedentary as the school year progressed. As an environmental intervention, the stand-biased desks mitigated this increase in sedentary behavior, particularly for those students who began the school year as highly sedentary.

Children become more sedentary as the school year progresses. At the start of the school year, the entire sample were sedentary for 61.2% of classroom time (3rd graders 52.8%; 4th graders 62.7%; 6th graders 64.4%), with the remaining time spent in LPA (overall 32.4%; 3rd graders 35.8%; 4th graders 33.0%; 6th graders 30.1%) or MVPA (overall 6.4%; 3rd graders 11.4%; 4th graders 4.4%; 6th graders 5.5%). These data show similar albeit slightly lower sedentary time compared with Contardo Ayala et al. (2016) who showed that students aged 11–12 years spend 69% of their classroom time sitting based on accelerometry data [15]. By the end of the school year, the current data showed that the amount of time sedentary increased as all students were more sedentary during classroom time. This time was reallocated from both LPA and MVPA. While the majority of active time in the classroom is sporadic, rather than sustained activity, this movement is still important for the growth, development and health of children. We hypothesize that the increase in sedentary behavior that was seen throughout the school year occurred partially because students were acculturated into the rules and structure of the classroom as the year progressed, which promoted limited movement to provide minimal distractions for learning in the classroom. Further research should explore reasons for the decreasing activity across the academic year, which may include seasonal variation in physical activity [28,29].

These data showed relatively minor changes in activity and sedentary behavior for participants overall as a result of the introduction of stand-biased desks. This contradicts earlier research that showed positive impacts of stand-biased desks in the classroom. Shorter interventions have shown success. Hinckson et al. (2013) reported that classrooms with standing workstations decreased sitting time by about 30 min more than the control classroom and increased standing by approximately 40 min while the control classroom decreased standing by about 20 min over the course of a full day [30]. A recent longer-term study that incorporated three desks into each classroom [23], showed improvements in sitting time (−26 min/school day) and standing time (+26 min/day) in 18 fifth grade students, as assessed by ActivPal. Looking at the Baseline to Post I data from this study, we see positive influences on sedentary behavior and activity behavior as a result of using the stand-biased desks. While both groups (those children in a stand-biased and traditional sitting desks) showed an increase in percent of classroom time sedentary and a decrease in MVPA, the changes in sedentary behavior and PA were significantly less in the group using stand-biased desks than those students using traditional sitting desks (between group differences in change scores: sedentary behavior, *p* = 0.0334; LPA, *p* = 0.773; MVPA, *p* = 0.0004). Specifically, the students using stand-biased desks increased sedentary behavior by 1.9% (24.7 min) and decreased MVPA by 0.6% (6.7 min) of classroom time, while students using traditional desks increased sedentary behavior by 6.2% (70.1 min) and decreased MVPA by 3.6% (39.6 min) of classroom time. The data from this present study and the data from Hinckson et al. [30] and Verloigne et al. [23] show that stand-biased desks can have a positive impact on the sedentary behavior/activity profile of students. However, as the study was lengthened, these positive results were diminished, suggesting that a simple environmental intervention is not sufficient for long-term behavior change in the classroom. Contardo Ayala et al. showed a significant reduction in classroom sitting time as a result of an 8-month intervention that included the use of stand-biased desks, training for teachers and pedagogical strategies. The results showed no significant between group differences (intervention vs. control) for total sitting time, but reduced time in prolonged (>10 min) bouts of sitting in the intervention group compared with the control group (18 fewer minutes in sitting bouts > 10 min in intervention vs. control group) [18]. Together, our data and those of Contardo Ayala [15] suggest that simply placing a standing desk in a classroom for children to use may promote healthy movement patterns for a short time. However, given that there are other influences on sedentary behavior and activity, including other children in the classroom, the grade or maturation of the student, the classroom teacher and their attitude towards stand-biased desks, and the curriculum, additional intervention strategies are needed to sustain healthy movement patterns in the classroom over the course of the school year.

The levels of sedentary behavior at the start of the school year highly influenced students’ response to desk type. Those students who were less active at the start of the year became more active in response to the standing desk compared with students in the traditional desk, while those students who were more active at the start of the academic year increased their sedentary behavior with the use of the stand-biased desks compared with students in the traditional desks. Figure 2 depicts the changes in sedentary behavior from baseline to follow-up. The sedentary behavior of the traditional desk group ranged from 30–80% of classroom time sedentary at baseline to 40–80% sedentary at follow-up, while the stand-biased desk group ranged from 30–80% of classroom time sedentary at baseline to 40–75% sedentary at follow-up, reinforcing that the most sedentary stand-biased desk users became less sedentary. It is for these students, who were more sedentary at the start of the year, that the use of stand-biased desks was able to minimize the increase in sedentary behavior. It appears that students using the stand-biased desks began to conform to uniform levels of activity during the academic year. If the educators recognize that children become more similar in their activity and sedentary behavior levels in the classroom and implement strategies to increase activity in the classroom, this could benefit all students in the class.

There are a number of limitations to be considered when reflecting on these results. Only one elementary school was included in this study, which limits the generalizability of the results. While the elementary school was culturally and economically diverse, there was limited diversity in education levels of the parents and the children were generally active, with more children meeting the physical activity recommendations than the national average [31]. Secondly, there were a number of children who did not complete the accelerometer assessment or wear log. However, we were able to account for this in our analysis. Despite these limitations, there were a number of strengths that are noteworthy. All waking hours were assessed with the accelerometer, providing us with information on whether they met or did not meet the PA recommendations. Second, there were multiple grades included and all classrooms within a grade were included. Including all classrooms within a grade allowed the student to remain at a standing desk despite switching classrooms for various subjects, therefore increasing the total daily exposure participants received of the intervention stimulus. Finally, the within classroom design was used to minimize teacher influence. Research has shown that the teacher is highly influential on students learning and attitudes [24]. While teacher influence was not measured in this study, if the teachers in the intervention classrooms were extremely positive about the use of standing desks, this could positively skew the results by providing an interventional tool that was not planned in the study design.

## 5. Conclusions

While school is only one opportunity of many for children to be sedentary, schools can provide opportunities for activity and help students to develop lifelong skills and knowledge of the physical, mental and emotional benefits of leading an active lifestyle. These data provide insight into the influence of an environmental intervention on sedentary behavior in schools and that this simple intervention can help to positively influence the sedentary and activity behaviors of the most sedentary children. However, in many schools around the world, there is a culture of sitting that is learned early in elementary school. Once children enter first grade, they are introduced to sitting desks and the time at those desks increases with increasing age. Additionally, when students are at the desks, they are often encouraged to remain sitting or sit still. These behaviors are learned and practiced throughout their educational career. Therefore, it may take more than one year of exposure to a stand-biased desk to reverse years of learned behavior.

## Figures and Tables

**Table 1:** Study demographics (*n* = 99).

Characteristics	Total Sample		3rd Grade		4th Grade		6th Grade	
	(*n* = 99, 100%)		(*n* = 22, 22.2%)		(*n* = 36, 36.4%)		(*n* = 41, 41.4%)	
	MEAN	SD	MEAN	SD	MEAN	SD	MEAN	SD
Age (Year)	10.2	1.4	8.5	0.5	9.6	0.6	11.7	0.5
Height (cm)	146.0	29.3	134.8	7.3	146.3	46.5	152.0	7.4
Weight (kg)	37.0	9.9	29.3	4.3	34.1	8.9	43.8	8.7
BMI Percentile ^a^	53.8	[26.5–74.9]	55.4	[41.1–68.5]	42.8	[28.2–73.1]	60.8	[19.0–86.1]
Male ^b^	56	57.1	15	68.2	15	41.7	26	65.0
Race ^b^								
White	69	79.3	18	90	21	67.74	30	83.3
Black/African-American	3	3.5	1	5	2	6.45	0	0.0
Asian	8	9.2	1	5	4	12.9	4	11.1
Mixed Race	7	8.1	0	0.0	4	12.9	2	5.6
Hispanic ^b^	8	9.4	2	10	1	3.23	5	14.7
Family Income ^b^								
Under $49,999	12	14.0	0	0.0	7	23.3	5	14.3
$50,000–$99,999	20	23.3	5	23.8	8	26.7	7	20.0
$100,000–$149,999	23	26.7	6	28.6	5	16.7	12	34.3
$150,000–$199,999	10	11.6	3	14.3	4	13.3	3	8.6
$200,000 or more	21	24.4	7	33.3	6	20.0	8	22.9
Valid Time Monitor Worn (h/d)	11.2	2.8	10.8	2.5	11.2	2.8	11.3	3.1
Average 60 Min of MVPA Per Day ^b^	48	53.3	14	70.0	14	41.2	20	55.6
Average 30 Min of MVPA During School Time ^b^	48	50.5	14	70.0	13	37.1	21	52.5

Note. ^a^ = Median and Interquartile Range presented instead of Mean and Standard Deviation. ^b^ = Frequency and Percentages are presented instead of Mean and Standard Deviation.

**Table 2:** Physical activity and sedentary behavior characteristics during classroom in those using a sitting desk versus a stand-biased desk (Mean (SD)).

MEASURES	Baseline			Post I			Post II		
	Sit-Stand	Stand-Sit	P_1_	Sit-Stand	Stand-Sit	P_2_	Sit-Stand	Stand-Sit	P_3_
	(*n* = 39)	(*n* = 58)		(*n* = 36)	(*n* = 47)		(*n* = 37)	(*n* = 52)	
**School Day**									
Average 30 Min of MVPA ^a^	25 (64.1%)	23 (41.1%)	0.027	15 (41.7%)	19 (40.4%)	0.909	15 (40.5%)	21 (42.0%)	0.891
3rd Grade	10 (100.0%	4 (40.0%)	0.011	9 (90.0%)	6 (66.7%)	0.303	6 (66.7%)	7 (70.0%)	1.000
4th Grade	8 (61.5%)	5 (22.7%)	0.033	6 (60.0%)	10 (52.6%)	1.000	5 (41.7%)	8 (42.1%)	0.981
6th Grade	7 (43.8%)	14 (58.3%)	0.520	0 (0.0%)	3 (15.8%)	0.234	4 (25.0%)	6 (28.6%)	1.000
**Classroom Time**									
Wear Time (min/day)	237.9 (20.2)	238.9 (26.3)	0.522	236.2 (34.8)	241.7 (32.5)	0.486	233.2 (22.0)	238.8 (26.4)	0.322
3rd Grade	225.6 (4.7)	213.2 (22.7)	0.105	226.7 (6.2)	225.6 (4.2)	0.647	224.4 (9.2)	220.8 (4.7)	0.277
4th Grade	227.3 (10.4)	228.2 (8.6)	0.820	225.9 (7.9)	232.6 (10.6)	0.178	227.0 (11.5)	226.3 (12.7)	0.899
6th Grade	251.6 (22.0)	258.7 (21.5)	0.315	246.0 (47.5)	256.5 (44.1)	0.508	241.3 (28.1)	256.9 (28.9)	0.108
Sedentary behavior (min/day)	122.7 (40.9)	136.2 (36.7)	0.113	132.8 (43.3)	139.8 (38.7)	0.464	151.8 (27.8)	152.5 (36.1)	0.930
3rd Grade	78.5 (26.2)	109.0 (14.9)	0.005	92.7 (14.9)	105.6 (22.3)	0.154	138.1 (17.8)	118.8 (13.8)	**0.017**
4th Grade	109.6 (19.2)	134.7 (27.2)	0.021	112.6 (26.0)	125.0 (27.2)	0.352	145.6 (26.2)	141.3 (21.3)	0.669
6th Grade	157.7 (21.5)	148.6 (43.2)	0.382	165.4 (33.9)	167.6 (33.4)	0.847	162.6 (30.1)	177.0 (35.6)	0.203
LPA (min/day)	83.4 (14.1)	85.4 (23.2)	0.614	83.4 (26.0)	84.8 (23.6)	0.810	73.8 (18.8)	78.0 (20.9)	0.359
3rd Grade	91.4 (10.8)	88.5 (18.8)	0.683	97.2 (7.2)	92.7 (15.9)	0.446	79.7 (23.4)	92.1 (11.8)	0.181
4th Grade	91.5 (10.3)	85.5 (22.0)	0.340	90.8 (29.7)	88.4 (17.9)	0.822	73.6 (16.3)	75.9 (14.3)	0.729
6th Grade	73.9 (11.9)	84.1 (26.3)	0.104	71.9 (28.0)	78.1 (29.2)	0.528	70.6 (17.4)	72.9 (25.7)	0.753
MVPA (min/day) ^b^	26.9 [16.5–36.4]	15.6 [6.1–22.6]	0.001	16.1 [8.7–32.1]	15.5 [6.6–24.0]	0.529	6.8 [4.0–9.9]	6.5 [8.7–11.1]	0.683
3rd Grade	60.3 [35.7–70.6]	14.0 [6.7–19.8]	0.003	35.6 [30.0–44.1]	26.1 [19.9–31.9]	0.071	5.3 [3.6–9.8]	10.8 [5.1–12.8]	0.222
4th Grade	23.2 [16.5–31.5]	5.1 [2.6–11.0]	0.002	17.2 [16.8–27.0]	18.1 [13.3–26.0]	0.618	6.9 [4.8–8.6]	5.8 [3.3–11.7]	0.694
6th Grade	20.6 [10.8–27.9]	22.5 [15.6–30.5]	0.452	8.7 [5.3–10.8]	7.0 [5.3–14.7]	1.000	6.8 [3.2–10.1]	6.5 [3.8–10.1]	0.843

Note. *p*-values obtained from independent samples *t*-test. ^a^ = Frequency and Percentages are presented instead of Mean (SD); *p*-value obtained from Fisher’s Exact Test. ^b^ = Median and Interquartile Range presented instead of Mean (SD); *p*-value obtained from Wilcoxon Rank Sum Test. P_1_ = comparing groups at Baseline. P_2_ = comparing groups at Post I. P_3_ = comparing groups at Post II. Sit-Stand group used a sitting desk from baseline (September) to Post 1 (December) and a stand-biased desk from January to April (Post II). Stand-Sit: used a sitting desk at baseline, standing desk from baseline (September) to Post I and a sitting desk from January to April (Post II). Baseline and Post I included five data collection days, Post II included four data collection days. Students attended school for 34.4 h/week.

**Table 3:** Mixed effects models to predict sedentary behavior and physical activity during classroom time at follow-up based on desk allocation and baseline levels.

Variables	Sedentary Behavior			LPA			MVPA *		
	EST	SE	P	EST	SE	P	EST	SE	P
Intercept	49.222	4.753	<0.001	16.584	3.252	<0.001	0.601	0.229	0.010
Male	−5.108	1.263	<0.001	3.750	1.179	0.002	0.257	0.092	0.007
Grade (REF: 6th)			<0.001			<0.001			<0.001
3rd Grade	−7.951	1.693	<0.001	6.283	1.515	<0.001	0.587	0.116	<0.001
4th Grade	−9.229	1.401	<0.001	6.132	1.333	<0.001	0.673	0.103	<0.001
Time	1.020	0.671	0.133	−2.064	0.626	0.002	−0.194	0.063	0.003
Standing Desk	7.717	3.685	0.040	3.508	2.708	0.199	0.480	0.139	0.001
Baseline Measure	0.276	0.066	<0.001	0.371	0.090	<0.001	0.307	0.072	<0.001
Standing Desk × Baseline Measure	−0.132	0.059	0.029	−0.083	0.082	0.314	−0.405	0.081	<0.001

* Natural log transformation was used for MVPA to normalize the data.
